# Coping With Negative Stereotypes Toward Older Workers: Organizational and Work-Related Outcomes

**DOI:** 10.3389/fpsyg.2019.00649

**Published:** 2019-03-22

**Authors:** Rita Chiesa, Sara Zaniboni, Dina Guglielmi, Michela Vignoli

**Affiliations:** ^1^Department of Psychology, University of Bologna, Bologna, Italy; ^2^Department of Education Studies, University of Bologna, Bologna, Italy; ^3^Department of Psychology and Cognitive Sciences, University of Trento, Rovereto, Italy

**Keywords:** older workers, age-related stereotypes, identification with the organization, occupational self-efficacy, psychological engagement, development opportunities for workers

## Abstract

The current study aims to test a moderated-mediation model in which occupational self-efficacy determines the indirect effect of negative stereotypes about older workers in the organization both on psychological engagement in the work domain and on attitudes toward development opportunities through identification with the company. The survey involved 1,501 Italian subjects aged over 50 who were employed by a major large-scale retailer. Consistently with the Social Identity Theory and the Social Exchange Theory, results showed that the perception of negative stereotypes about older workers in the organization is associated with low identification with the company and, subsequently, with poor psychological engagement in the work domain and with attitudes indicating very little interest in development opportunities. In addition, this association was found to be stronger in older workers with higher and medium levels of occupational self-efficacy. These findings suggest that organizations should discourage the dissemination of negative stereotypes about older workers in the workplace because they may lead to older workers’ disengagement from the work domain and their loss of interest in development opportunities.

## Introduction

Nowadays, population aging across the Western world is increasing the need to extend the participation of older people in the labor market. However, workplaces often present a number of barriers that might strongly impact on older employees’ motivation at work, willingness to learn and intention to retire (Billett et al., 2011; Kanfer et al., 2013; Hertel et al., 2013). Specifically, the literature about occupational stress (i.e., Adams et al., 2013) reports that older workers might experience different social stressors in their work environment stemming from ageism. Ageism is the “systematic stereotyping of and discrimination against older people because they are old” (Butler, 1989, p. 139). Age stereotypes, defined as beliefs and expectations regarding a worker, based on his or her age (Hamilton and Sherman, 1994; Posthuma and Campion, 2009), generally refer negatively to older workers as poorly productive, resistant to change, unable to learn new solutions, and less healthy in comparison with younger workers (i.e., Posthuma and Campion, 2009; Ng and Feldman, 2010; Dordoni and Argentero, 2015). According to the Social Identity Theory (SIT; Tajfel and Turner, 1986), negative stereotypes could be a source of unfavorable self-evaluation (Branscombe and Ellemers, 1998), as well as a threat to people’s desire to be considered positively (Finkelstein et al., 2013). Older workers may adopt different strategies to cope with ageism: some of them are individual strategies adopted to improve the older worker’s status as an individual, and others are collective strategies to improve the status of the group of older workers as a whole (Desmette and Gaillard, 2008). In this sense, disengaging, either physically or psychologically, from their own work and organization may be considered an individual strategy that will allow older workers to re-establish the perceived integrity of self (Chasteen and Cary, 2015). In a similar vein, consistently with the norm of reciprocity formulated by the Social Exchange Theory (Blau, 1964; Cropanzano and Mitchell, 2005), negative age stereotypes may encourage older employees to reduce the perceived imbalance between effort and reward by decreasing engagement in the work domain and in development opportunities. The current study intends to explore the impact of negative age stereotypes on psychological engagement in the work domain and positive attitude toward development opportunities, hypothesizing a meditational effect of organizational identification moderated by occupational self-efficacy.

### The Consequences of Negative Stereotypes About Older Workers on Psychological Engagement in the Work Domain and on Attitudes Toward Development Opportunities

Employee engagement has been defined in many different ways (Saks, 2006) but, in any case, it has been found to be negatively related with age stereotypes (i.e., Gaillard and Desmette, 2008; James et al., 2013). Given that individuals express their preferred selves through work engagement (Kahn, 1990), work engagement may be influenced by the extent to which the organization is integrated into the self-definition of individuals.

Older people can adopt many coping strategies to protect their self-view from age stigma (Chasteen and Cary, 2015), indeed, according to the SIT (Tajfel and Turner, 1986), people define themselves in terms of their social group memberships striving to maintain a positive self-image. Disengagement strategies are aimed to abandon the stigmatized group or, alternatively, to psychologically escape from the stigma by reducing the centrality of the stigmatized identity to self. According to Desmette and Gaillard (2008) and Gaillard and Desmette (2008), this is what happens when older workers decide to retire in order to withdraw physically from their stigmatized group, or to psychologically disengage from their work. By detaching their self-worth from external feedback or from outcomes in the work domain, older workers make their feelings of self-worth independent from success or failure in that domain (Major and Schmader, 1998). Similarly, Gaillard and Desmette (2010) observed that older workers who were exposed to negative age-related stereotypic information were more willing to retire and less interested in learning and developing, than those who were exposed to positive age-related stereotypic information. The current study assumes that negative age stereotypes impact on psychological engagement in work domain and attitudes toward training and development because they reduce the organizational identification. Indeed, given that organizational identity is a specific form of social identity, and that organizational identification represents the strength of this identity (Haslam, 2004), we expect decreasing organizational identification to become a coping strategy against negative age stereotypes.

### Negative Stereotypes About Older Workers and Organizational Identification: The Moderating Role of Occupational Self-Efficacy

Scholars agree that the choice of a specific strategy to cope with stereotypes may be affected by individual attributes (i.e., Berjot and Gillet, 2011; Chasteen and Cary, 2015). In particular, self-efficacy is considered a personal resource that may facilitate dealing with potentially aversive events because it refers to the individual’s ability and willingness to exercise control (Bandura, 1977). Hence, its role in moderating the negative effect of potential work environment stressors has been the focus of interest in occupational stress literature (Stetz et al., 2006). Given that the efficacy belief system is not a global trait but a differentiated set of self-beliefs linked to distinct functional domains (Bandura, 2005), this study focuses on a domain-specific measure of self-efficacy, namely occupational self-efficacy, the competence people feel concerning their ability to successfully fulfill the tasks required by their job role (Rigotti et al., 2008). The aim is to explore the moderating role of self-efficacy in determining the adoption of a disengagement strategy in response to organizational age stereotypes. Previous studies (Fletcher et al., 1992) have provided evidence that occupational self-efficacy is more negatively related to job stress among workers aged 50 and older, than among younger workers. We expect age stereotypes to decrease organizational identification, and to subsequently reduce both psychological engagement in the work domain and positive attitudes toward development opportunities. Older workers with high occupational self-efficacy will perceive themselves as able to do their job (Schyns and Von Collani, 2002) and, consequently, they will likely feel less similar to other older employees who are stereotypically considered as ineffective at work. The perception of similarities and differences between self and other in-group members is based on self-definition as either a unique individual or as an interchangeable group member (i.e., Simon et al., 1995). Ellemers and Van Rijswijk (1997) found that perceived intragroup heterogeneity is associated with an individual response to unfavorable intergroup comparison. Consistently, people who perceive themselves as dissimilar to the stereotypical image of older workers will more likely adopt a self-protection strategy that entails decreasing their identification in an organization that is a source of negative identity.

Moreover, research indicates that individuals tend to select interactions with others who provide self-confirming feedback. It means that employees with a positive self-view will seek an organization that provides positive feedback about their performance (Swann, 1990), and they will detach from one that applies negative stereotypes about them. Finally, the moderating role of self-efficacy in the relationship between negative age stereotypes and older workers’ organizational identification is understandable in the light of the Social Exchange Theory’s perspective too. The Social Exchange Theory (Blau, 1964; Cropanzano and Mitchell, 2005) defines social exchanges as a series of interactions over time regulated mainly by the norm of reciprocity. Consistently, Saks (2006) argued that employees who receive socio-emotional resources from their organization to meet their social and self-esteem needs feel obliged to repay the organization with high levels of engagement. Conversely, when the organization fails to provide a good fit between socio-emotional resources and individual needs, employees are more likely to withdraw and disengage themselves from their work roles in order to restore the effort-reward balance. Thus, reciprocal interdependence provides a theoretical foundation to explain why employees become engaged in their work and organization. Obtaining affective rewards (emotional satisfaction) and supporting personal identity become more and more important during later adulthood (Carstensen, 1998; Kanfer and Ackerman, 2004). Hence, age stereotypes may create a strong misfit between older workers’ expectations to be rewarded and the actual situation in the organization (i.e., Bal et al., 2015). In line with these assumptions, Armstrong-Stassen and Ursel (2009) found that older workers who perceived that their organization valued their contribution and cared about their well-being expressed higher levels of career satisfaction and a greater intent to continue working, than those who perceived little organizational support. In a similar vein, Rabl and Triana (2013) suggested that older workers who experienced exclusion or were disadvantaged because of their age decreased their affective organizational commitment in order to minimize the net loss of resources resulting from the perceived lack of acknowledgment of personal accomplishments.

We expect a higher perception of the worker’s own value to correspond to a higher expected organizational reward. Hence, older workers who possess higher occupational self-efficacy will consider the presence of negative stereotypes that devalue older worker’s contributions to the organization a more serious violation of the norm of reciprocity, with a subsequent stronger negative effect on their identification with the company.

Accordingly, we propose the following hypothesis:

Hypothesis 1 – Occupational self-efficacy moderates the relationship between negative stereotypes about older workers and their identification with the company; therefore, the perception of negative stereotypes about older workers in the organization will be more negatively related to the identification with the company in older workers with higher self-efficacy, compared to their colleagues with lower self-efficacy.

In addition, the current study intends to explore the meditational role of organizational identification in the relationship between age stereotypes and, respectively, psychological engagement in the work domain and attitudes toward development opportunities at different levels of occupational self-efficacy. There is evidence (Murray et al., 2015; Dai and Qin, 2016) that high identifiers within the organization are more involved in achieving organizational goals by engaging with their work. There is also evidence that individuals who have a strong psychological bond with their organization are more inclined to expend their time and energies in acquiring new skills and expertise in order to contribute to the growth and development of the organization (i.e., Chughtai and Buckley, 2010). Hence, we expect that negative age stereotypes may cause psychological disengagement in the work domain and negative attitudes toward development opportunities especially in older workers with a high level of occupational self-efficacy because they will have a lower level of organizational identification.

Consequently, we propose the following hypothesis:

Hypothesis 2 – The indirect effect of negative stereotypes about older workers on their psychological engagement in the work domain (H2a) and on their attitudes toward development opportunities (H2b) through identification with the company depends on occupational self-efficacy. Hence, for older workers with higher occupational self-efficacy, the perception of negative stereotypes about older workers in the organization will have a larger negative impact on their identification with the company and, in turn, on psychological engagement in the work domain, and on the attitudes toward development opportunities (moderated-mediation model).

**FIGURE 1:**
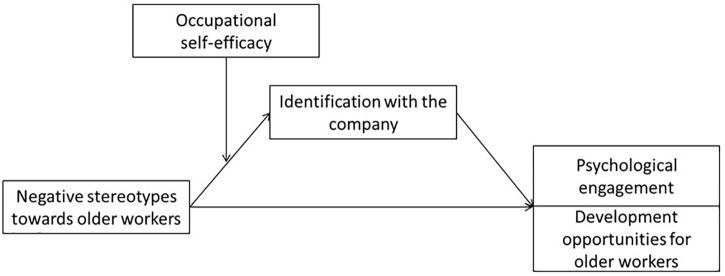
Moderated-mediation models.

The conceptual model framing the study variables in a moderated mediation relationship is reported in Figure 1.

## Materials and Methods

### Participants and Procedure

Participants included in the study were 1,501 Italians aged over 50, employed by a supermarket chain. Overall, 559 completed the variables of interest in this study (response rate 37.24%). The age of participants was chosen based on the age interval conventionally used by studies on older workers (Zaniboni, 2015). The sample comprised 69% male workers (*n* = 387), and the mean age was 53.64 years (*SD* = 2.57; range: 50–65). Regarding the job type held by participants, 25.4% (*n* = 142) were office administration clerks, 64.4% (*n* = 371) were sales officers, and 8.2% (*n* = 46) did not provide this information. The average organizational tenure was 19.79 years (*SD* = 9.54), and 67% (*n* = 375) of the participants were working full time.

Data was collected through a cross-sectional self-reported survey that was distributed by e-mail. An anonymous link to the questionnaire was generated by the Qualtrics platform^1^ and sent by e-mail. At the same time, the e-mail informed them that participation in the survey was entirely voluntary, and that all the answers were anonymous. Given the anonymity of responses, the consent of participants was given by completing the survey after they were provided with sufficient information about the study, in accordance with the Italian privacy law. Procedural remedies were used to reduce common method variance (Podsakoff et al., 2003). Respondents were informed that there were neither right nor wrong answers, and they were asked to answer the questions as honestly as possible (Podsakoff et al., 2003). Moreover, the model included an interaction effect based on the suggestion that relations between negative stereotypes about older workers and identification with the company were different for older workers with high and low occupational self-efficacy. Thus, the hypothesized model was probably not part of the respondents’ cognitive map, and this reduced the threat of respondents “guessing” (Harrison et al., 1996; Chang et al., 2010).

### Measures

#### Negative Stereotypes About Older Workers

We used the 13-items in an Italian version of Henkens’s (2005) scale validated by Chiesa et al. (2016). Respondents were asked to indicate to what extent they agreed with the statements presented, which referred to their organization’s negative beliefs about workers aged 50 years and older. A sample item was “My organization thinks that… older workers are less productive than younger workers.” The response scale ranged from 1 (*strongly disagree*) to 5 (*strongly agree*). The coefficient alpha in this study was 0.76.

#### Occupational Self-Efficacy

We measured the perceived self-efficacy related to the work domain with the eight items of the Italian version (Di Fabio and Taralla, 2006) of the Occupational Self-Efficacy Scale (short form) (Schyns and Von Collani, 2002; Rigotti et al., 2008). A sample item was “Whatever comes my way in my job, I can usually handle it.” The original six-point response scale was adjusted into a five-point one ranging from 1 (*not at all true*) to 5 (*completely true*). The coefficient alpha in this study was 0.87.

#### Identification With the Company

We adapted three items from Farth et al. (1997) to describe employees’ identification with the company, and their willingness to protect the corporate reputation by making suggestions to improve the organization’s functional features. A sample item was “I am willing to stand up to protect the reputation of the company.” Responses were obtained in addition to adapting the original seven-point scale to a five-point one where it ranged from 1 (*strongly disagree*) to 5 (*strongly agree*). The coefficient alpha in this study was 0.76.

#### Psychological Engagement in the Work Domain

It was measured by six items adapted from Major and Schmader (1998; Schmader et al., 2001) that refer to “a detachment of self-esteem from external feedback or outcomes in a particular domain, such as the feelings of self-worth, are not dependent on the successes or failures in that domain” (Major and Schmader, 1998; p. 220). A sample item was “Doing my job well is very important to me.” These items were rated on a five-point scale ranging from 1 (*strongly disagree*) to 5 (*strongly agree*). The coefficient alpha in this study was 0.75.

#### Attitudes Toward Development Opportunities for Older Workers

We used three ad hoc items to assess the extent to which respondents evaluate the organization’s initiatives for the development older workers as useful. Participants rated their perception of the level of opportunities of “counseling for career development,” “training,” and “campaigns for recruiting older workers” on a five-point Likert scale ranging from 1 (*totally useless*) to 5 (*totally useful*). The coefficient alpha in this study was 0.76.

## Results

Means, standard deviations, correlations, and alpha reliabilities of the variables are presented in Table 1. Negative stereotypes about older workers resulted in a negative correlation with occupational self-efficacy (*r* = -0.31, *p* < 0.01), identification with the company (*r* = -0.28, *p* < 0.01), psychological engagement in the work domain (*r* = -0.29, *p* < 0.01), and attitudes toward development opportunities (*r* = -0.18, *p* < 0.01). Conversely, occupational self-efficacy was positively correlated with identification with the company (*r* = 0.39, *p* < 0.01), psychological engagement in the work domain (*r* = 0.31, *p* < 0.01) and attitude toward development opportunities (*r* = 0.20, *p* < 0.01). Moreover, identification with the company was positively correlated both with psychological engagement in the work domain (*r* = 0.44, *p* < 0.01) and with attitudes toward development opportunities (*r* = 0.20, *p* < 0.01). We used the covariance matrix as input and maximum likelihood as estimation method to perform a CFA on the variables considered in our model. The CFA 1-factor model [χ^2^ (527) = 12530.54, *p* = 0.00; RMSEA = 0.16; NNFI = 0.37; CFI = 0.41] was compared to the CFA 5-factor model [χ^2^ (517) = 3115.67, *p* = 0.00; RMSEA = 0.08; NNFI = 0.82; CFI = 0.83]. The chi-square difference test was significant [Δχ^2^ (10) = 9414.87, *p* < 0.01]; thus, the model with five factors was preferred. Moreover, we used specification search (Leamer, 1978) to improve the fit of the 5-factor model. Particularly, following modification indices, some measurement errors were correlated with the scale of negative stereotypes about older workers. The modified model showed acceptable fit indices [χ^2^ (511) = 2073.94, *p* = 0.00; RMSEA = 0.06; NNFI = 0.90; CFI = 0.91].

**Table 1:** Means, standard deviations, and intercorrelations among study variables.

	*M*	*SD*	1	2	3	4	5
1. Negative stereotypes toward older workers	2.44	0.62	(0.76)				
2. Occupational self-efficacy	4.09	0.72	-0.31^∗∗^	(0.87)			
3. Identification with the company	3.72	0.87	-0.28^∗∗^	0.39^∗∗^	(0.76)		
4. Psychological engagement	3.06	0.92	-0.29^∗∗^	0.31^∗∗^	0.44^∗∗^	(0.75)	
5. Development opportunities for older workers	3.48	1.14	-0.18^∗∗^	0.20^∗∗^	0.20^∗∗^	0.15^∗∗^	(0.76)


**N* = 559. Cronbach’s alpha in brackets on the diagonal. ^∗^*p* < 0.05; ^∗∗^*p* < 0.01.*

PROCESS macro (Hayes, 2012) was used to test our moderated-mediation model where the interaction between negative stereotypes about older workers (independent variable) and occupational self-efficacy (moderator) affects identification with the company (mediator) which, in turn, affects psychological engagement in the work domain and attitudes toward development opportunities (outcomes). We specified 10,000 bootstrap samples to obtain robust estimates of standard errors and confidence intervals (Preacher et al., 2007), namely centered, independent, and moderator variables.

Table 2 reports the results of the moderated-mediation models tested^2^. Negative stereotypes about older workers (*B* = -0.22, *p* = 0.00) and the interaction between negative stereotypes about older workers and occupational self-efficacy (*B* = -0.18, *p* = 0.00) negatively affected identification with the company. Instead, occupational self-efficacy positively affected identification with the company (*B* = 0.42, *p* = 0.00). According to our Hypothesis 1, occupational self-efficacy moderated the relationship between negative stereotypes about older workers and identification with the company. Hence, the presence of negative stereotypes about older workers in the organization was more negatively related to identification with the company in older workers with higher occupational self-efficacy, compared to their colleagues with lower occupational self-efficacy. Thus, Hypothesis 1 was confirmed.

**Table 2:** Results of the moderated-mediation models.

	Identification with the company (M)		Psychological engagement (Y_1_)		Development opportunities for older workers (Y_2_)	
			
Variable	*Coefficient*	*SE*	*Coefficient*	*SE*	*Coefficient*	*SE*
Negative stereotypes toward older workers (X)	-0.22^∗∗^	0.06	-0.28^∗∗^	0.06	-0.24^∗∗^	0.08
Occupational self-efficacy (W)	0.42^∗∗^	0.06				
Negative stereotypes toward older workers × occupational self-efficacy	-0.18^∗^	0.09				
Identification with the company (M)			0.41^∗∗^	0.04	0.21^∗∗^	0.06

Model of M summary	*R*^2^ = 0.19^∗∗^					

Model of Y_1_ summary			*R*^2^ = 0.22^∗∗^			

Model of Y_2_ summary					*R*^2^ = 0.06^∗∗^	

*Conditional indirect effect of negative stereotypes toward older workers (X) on psychological engagement (Y) through identification with the company (M) at values ofoccupational self-efficacy (W)*						

**Occupational self-efficacy**	**Effect**		**Boot SE**		**Boot 95% CI**	

Lower	-0.03		0.04		-0.12 – 0.04	
Medium	-0.09		0.03		-0.14 – -0.04	
Higher	-0.14		0.03		-0.21 – -0.08	

*Conditional indirect effect of negative stereotypes toward older workers (X) on development opportunities for older workers (Y) through identification with the company (M) at values of occupational self-efficacy (W)*						

**Occupational self-efficacy**	**Effect**		**Boot SE**		**Boot 95% CI**	

Lower	-0.02		0.02		-0.07 – 0.02	
Medium	-0.05		0.02		-0.10 – -0.02	
Higher	-0.08		0.03		-0.14 – -0.03	


**N* = 559. ^∗^*p* < 0.05; ^∗∗^*p* < 0.01.*

The two models of the dependent variables showed that identification with the company significantly and positively affected psychological engagement in the work domain (*B* = 0.41, *p* = 0.00) and the attitude toward development opportunities (*B* = 0.21, *p* = 0.00). According to Hypothesis 2, the indirect effects of negative stereotypes about older workers, respectively, on psychological engagement in the work domain (H2a) and on attitudes toward development opportunities (H2b) through identification with the company depended on the occupational self-efficacy level. The lower part of Table 2 reports critical values of conditional indirect effects (Hayes, 2012). Results indicate that the indirect effects of negative stereotypes about older workers on psychological engagement in the work domain and on attitudes toward development opportunities through identification with the company were significant at higher and medium levels of occupational self-efficacy. In particular, the effects were significant and negative for workers perceiving higher levels of occupational self-efficacy (for psychological engagement, -0.14, CI = [-0.21, -0.08]; for attitude toward development opportunities, -0.08, CI = [-0.14, -0.03]) and medium levels of occupational self-efficacy (for psychological engagement, -0.09, CI = [-0.14, -0.04]; for attitudes toward development opportunities, -0.05, CI = [-0.10, -0.02]). They were not significant for workers perceiving lower levels of occupational self-efficacy (for psychological engagement, -0.03, CI = [-0.12, 0.04]; for attitude toward development opportunities, -0.02, CI = [-0.07, 0.02]). Our Hypotheses 2a and 2b were thus confirmed.

**FIGURE 2:**
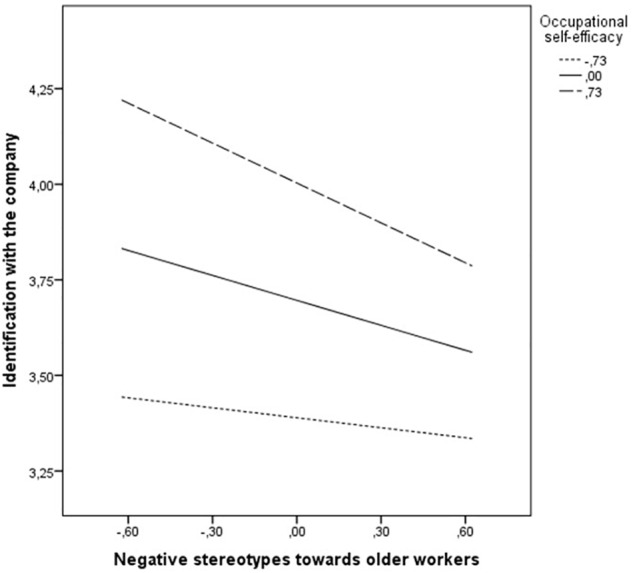
Workers’ self-efficacy and negative stereotypes toward older workers interact to affect the identification with the company. Lower occupational self-efficacy: 1 standard deviation below the mean (M – 1SD = 3.37). Medium occupational self-efficacy: the mean (*M* = 4.09). Higher occupational self-efficacy: 1 standard deviation above the mean (M + 1SD = 4.81).

Figure 2 provides more details regarding the effect of interaction between negative stereotypes about older workers and occupational self-efficacy on identification with the company, showing that the negative relationship between organizational negative stereotypes about older workers and identification with the company was stronger for higher and medium levels of occupational self-efficacy.

## Discussion

The aim of this study was to examine a moderated-mediation model in which the indirect effect of negative stereotypes about older workers regarding their psychological engagement in the work domain and their attitudes toward development opportunities through identification with the company depend on occupational self-efficacy. Results support the hypotheses indicating that the perception of negative stereotypes about older workers in the organization is negatively associated with the identification of the company and, in turn, with psychological engagement in the work domain and with attitudes toward development opportunities. In addition, they confirm that this relationship is stronger in older workers with higher and medium levels of occupational self-efficacy.

### Contributions to Theory and Research

Consistently with the SIT, which assumes that people may adopt different strategies to cope with negative stereotypes in order to protect their self-image or the overall image of their group (Desmette and Gaillard, 2008), our findings suggest that especially older workers with either medium or high levels of occupational self-efficacy are inclined to adopt a disengagement strategy that decreases organizational identification with negative consequences for psychological engagement in the work domain and for attitudes toward learning and development. This is probably due to the fact that they perceive themselves as quite dissimilar to the other older workers who are considered negatively by the organization and, therefore, they select an individual response to protect their positive sense of self. Conversely, the effect of negative age stereotypes on organizational identification is not significant for older workers with low occupational self-efficacy, perhaps because they may adopt different strategies to cope with age stereotypes.

This paper contributes to research on the Social Exchange Theory too. Our findings are of particular interest because they show the mediational effect of identification with the company in this relationship at different levels of occupational self-efficacy. As Edwards (2009) argued, employees embrace the organization’s values and goals as their own in exchange for acknowledgment of their contribution. Therefore, the presence in the workplace of negative stereotypes about older workers may be perceived as a lack of reciprocity that leads older employees to reduce their organizational identification and their investment in the work domain in order to limit the loss of resources (Rabl and Triana, 2013). This is especially true when the level of occupational self-efficacy is either medium or high because the perception of imbalance between invested and gained resources is higher in these cases.

When occupational self-efficacy was low, we did not observe any effect of negative age stereotypes on the identification with the company. Perhaps this is because our respondents justified the organization’s negative beliefs about them with their low ability to successfully fulfill the tasks required by their job. In this sense, negative age stereotypes could be considered self-confirming feedback that does not require overcoming the imbalance between efforts and rewards.

### Practical Implications

Human resources (HR) practices may offer resources and opportunities to prolong the work life of employees but, despite the increasing number of HR strategies designed to promote the use and retention of older workers, empirical evidence about their effectiveness is largely insufficient (Kooij et al., 2010). Indeed, little is known about older workers’ needs and the motives that can retain them at work. The general assumption is that people in their late careers are not likely to be thriving and their goals are less growth-oriented than younger workers’ goals. Hence, “age-friendly” HR strategies often aim to maintain older workers in their current functional level, rather than to encourage them to achieve new and challenging levels. However, recent studies have shown that older workers considered learning and development opportunities provided by the organizations as an interesting opportunity (Veth et al., 2015; Taneva et al., 2016). Our study suggests that in order to prevent disengagement from the work domain and loss of interest toward development opportunities, it is important to discourage the dissemination of negative stereotypes about older workers in the workplace. This is because the perception of an identity threat and the lack of reciprocity in the relationship with the organization may lead older workers to adopt work avoidance reactions that create a self-fulfilling prophecy for them. Despite an evident lack of studies that investigate how to decrease stereotypes about older workers in the workplace, some experimental studies suggest which kind of interventions organizations could implement to decrease older workers’ stereotypes. For example, Abrams et al. (2006) found that according to the intergroup contact theory, positive intergenerational contacts were associated with reduced prejudice and reduced in-group identification, suggesting that organizational intervention aimed to increase positive intergenerational contacts could diminish vulnerability to the stereotype threat among older people. More in general, age-inclusive HR practices demonstrated to foster the development of positive organizational age-diversity climate, which in turn is related with important outcomes as job crafting (Ingusci, 2018), company’s performance, employees’ commitment and turnover intentions (Kunze et al., 2011; Boehm et al., 2014).

### Limitations and Future Research

Although this study makes an important contribution, it also has some limitations that future research should address. First, the data available were cross-sectional and were acquired from self-reports; hence, they may have been affected by a common method variance (Podsakoff et al., 2003). As suggested by Podsakoff et al. (2003), procedural remedies were used to control and counteract the common method variance. In order to reduce evaluation apprehension and prevent response distortion, respondents’ anonymity was protected with respect to their employer, respondents were advised that there were neither right nor wrong answers, and they were asked to answer questions as honestly as possible. Furthermore, the variables were measured with validated scales that have already been used in the aging research field, which can mitigate measurement error and thereby decrease common method bias (Spector, 1987). In addition, a moderated relationship was considered, reducing the threat of respondents “guessing” patterns (Harrison et al., 1996; Chang et al., 2010). Finally, the results of the confirmatory factor analysis revealed that the 5-factor model provided a significantly better fit to the data rather than the 1-factor model, which can suggest that the common method bias is not a serious concern in this study. In any case, future research should repeat the study using a longitudinal or time-lagged research design, and by gathering data from multiple sources. Second, future research should explore the possibility that other personal resources, besides self-efficacy (e.g., work ability), can moderate relations between negative stereotypes about older workers, identification with the company, psychological engagement in the work domain and attitude toward development opportunities for older workers. Moreover, considering the importance of keeping older workers at work, retirement-related outcomes (e.g., retirement intentions) should be included in future research. Furthermore, only workers from one organization participated in this study; therefore, the results should be retested with different working populations and organizations to increase the external validity of the study. Finally, the current study involved only workers aged over 50, while it could be interesting to explore generational differences, among younger, middle-aged and older workers. Especially, some recent research (Ingusci, 2018) suggested that older workers may play an important role in motivating their middle-aged colleagues, supporting them in seeking and coping with new challenges, and encouraging them to produce new ideas.

## Conclusion

In conclusion, this study makes an important contribution by addressing a gap in research regarding the interaction between negative stereotypes about older workers in the organization and occupational self-efficacy in affecting identification with the company, psychological engagement in the work domain and attitude toward development opportunities. Specifically, we found that stereotypes about older workers are more negatively related to identification with the company and, therefore, to psychological job engagement in the work domain and to the attitude toward development opportunities when levels of occupational self-efficacy are at high and medium levels. We believe these findings have important implications for both practice and research, and we encourage future research on the relationship between age-related stereotypes, personal resources at work, and organizational and retirement outcomes in various jobs and industry types.

## Ethics Statement

Ethical approval was not required for this study in accordance with the national and institutional guidelines. In fact, data for this study were collected through a self-reported survey distributed by email. Participants received an anonymous link generated by Qualtrics platform (www.qualtrics.com) to complete the questionnaire.

## Author Contributions

RC and SZ conceptualized the model and chose the theoretical framework. RC defined the tools and collected the data. SZ performed the data analyses. RC, SZ, and MV wrote the first draft of the paper. DG revised the paper. All the authors gave final approval of the paper.

## Conflict of Interest Statement

The authors declare that the research was conducted in the absence of any commercial or financial relationships that could be construed as a potential conflict of interest.

## Footnotes

1 www.qualtrics.com

2 As requested by a reviewer, simple mediation models have been tested. The mediation effect of identification with the company on the relationship between negative stereotypes toward older workers and psychological engagement in the work domain/attitudes toward development opportunities is significant.
